# The Virulence Protein SopD2 Regulates Membrane Dynamics of *Salmonella*-Containing Vacuoles

**DOI:** 10.1371/journal.ppat.1001002

**Published:** 2010-07-15

**Authors:** Nina Schroeder, Thomas Henry, Chantal de Chastellier, Weidong Zhao, Aude-Agnès Guilhon, Jean-Pierre Gorvel, Stéphane Méresse

**Affiliations:** Centre d'Immunologie de Marseille-Luminy, CNRS UMR 6102, INSERM U631, Université de la Méditerranée, Parc Scientifique de Luminy, Marseille, France; Institut Pasteur, France

## Abstract

*Salmonella enterica* serovar Typhimurium is a Gram-negative bacterial pathogen causing gastroenteritis in humans and a systemic typhoid-like illness in mice. The capacity of *Salmonella* to cause diseases relies on the establishment of its intracellular replication niche, a membrane-bound compartment named the *Salmonella*-containing vacuole (SCV). This requires the translocation of bacterial effector proteins into the host cell by type three secretion systems. Among these effectors, SifA is required for the SCV stability, the formation of *Salmonella-*induced filaments (SIFs) and plays an important role in the virulence of *Salmonella*. Here we show that the effector SopD2 is responsible for the SCV instability that triggers the cytoplasmic release of a *sifA* ^−^ mutant. Deletion of *sopD2* also rescued intra-macrophagic replication and increased virulence of *sifA^−^* mutants in mice. Membrane tubular structures that extend from the SCV are the hallmark of *Salmonella-*infected cells. Until now, these unique structures have not been observed in the absence of SifA. The deletion of *sopD2* in a *sifA^−^* mutant strain re-established membrane trafficking from the SCV and led to the formation of new membrane tubular structures, the formation of which is dependent on other *Salmonella* effector(s). Taken together, our data demonstrate that SopD2 inhibits the vesicular transport and the formation of tubules that extend outward from the SCV and thereby contributes to the *sifA^−^* associated phenotypes. These results also highlight the antagonistic roles played by SopD2 and SifA in the membrane dynamics of the vacuole, and the complex actions of SopD2, SifA, PipB2 and other unidentified effector(s) in the biogenesis and maintenance of the *Salmonella* replicative niche.

## Author Summary

*Salmonella typhimurium* is a bacterial pathogen that causes diseases ranging from gastroenteritis to typhoid fever. This bacterium survives inside eukaryotic cells within a membrane-bound compartment, namely the *Salmonella*-containing vacuole. *Salmonella* injects proteins, named effectors, into the infected cell. These effectors change the biology of the infected cell and collectively support *Salmonella* replication and virulence. The effector SifA plays a key role in the bacterial vacuole stability and in the formation of membrane tubules that extend from the vacuole. Absence of SifA leads to the disruption of the vacuolar membrane and, therefore to the release of bacteria in the cytosolic compartment. Consequently, this mutant presents significant replication and virulence defects. Here, we show that an additional *Salmonella* effector, SopD2, is responsible for the membrane instability of the *sifA^−^* vacuole. In addition, we demonstrate that SopD2 acts as an inhibitor of vesicle transport from the vacuole and that it down-modulates the formation of tubular structures. These findings describe a role for SopD2 as an antagonist of SifA in terms of vacuolar membrane dynamics.

## Introduction

The virulence of *Salmonella enterica* serovar Typhimurium requires its intracellular replication within a membrane-bound compartment called the *Salmonella-*containing vacuole (SCV). This is achieved by the expression of a Type 3 Secretion System (T3SS-2) encoded by the *Salmonella* Pathogenicity Island 2 (SPI-2) that enables *Salmonella* to translocate bacterial effectors into the infected cell [1], [2], [3], [4], [5], [6]. More than 20 T3SS-2 effectors have been identified so far, but their molecular activities remain largely unknown [7]. Some of these T3SS-2 effectors display enzymatic activities [8], [9], [10], [11], [12] or target host proteins [13], [14].

The T3SS-2 effector SifA is required for the stability of the SCV membrane [15]. The vacuolar membrane instability results in the gradual release of *sifA^−^* bacteria into the host cytosplasm. As *Salmonella* is unable to replicate in macrophage cytosol [15], [16], this mutant presents a strong virulence defect in mice [15], [17]. *Salmonella*-infected cells are characterized by the presence of membrane tubules enriched in late endosomal markers, known as *Salmonella*-induced filaments (SIFs) [17], [18]. In addition, a recent study by Mota *et al.* shows that *Salmonella* recruits membrane from a TGN-derived Scamp3-containing pathway to induce the formation of Scamp3-positive tubules, comprising the SIFs and the *Salmonella*-Induced Scamp3 Tubules (SISTs) [19]. While SISTs are devoid of late endocytic markers, SIFs are positive for both late endocytic markers and Scamp3 [19]. Both SIFs and SISTs require SifA for their development.

Upon translocation, SifA and the T3SS-2 effector PipB2 localize to the cytosolic face of the SCV and recruit the mammalian proteins SKIP and kinesin-1, respectively [13], [14]. By interacting with kinesin-1 [13], SKIP promotes the transport of SCV-derived vesicles towards the cell periphery [20]. A *sifA^−^* mutant is unable to recruit SKIP and is therefore defective in the transport of these membrane vesicles [21]. As a consequence, a *sifA^−^* SCV accumulates high levels of kinesin-1 [13]. The disruption of the *sifA* ^−^ vacuolar membrane is not due to the PipB2-mediated accumulation of kinesin-1 [14], [22] but requires the lipase activity of the T3SS-2 effector SseJ [23]. A *sifA^−^sseJ^−^* strain resides in a stable vacuole but displays the same intracellular replication defect as a *sifA^−^* mutant [24]. In this paper, we show that deletion of *sopD2* in a *sifA^−^* mutant stabilizes the SCV and restores intra-macrophagic replication and virulence. It induces the formation of LAMP1- and Scamp3-negative membrane tubules, which constitute a so far undescribed tubular network that forms independently of SIFs and SISTs.

## Results

### A *sifA* ^−^ *sopD2* ^−^ mutant strain resides in a stable SCV

**Figure 1:**
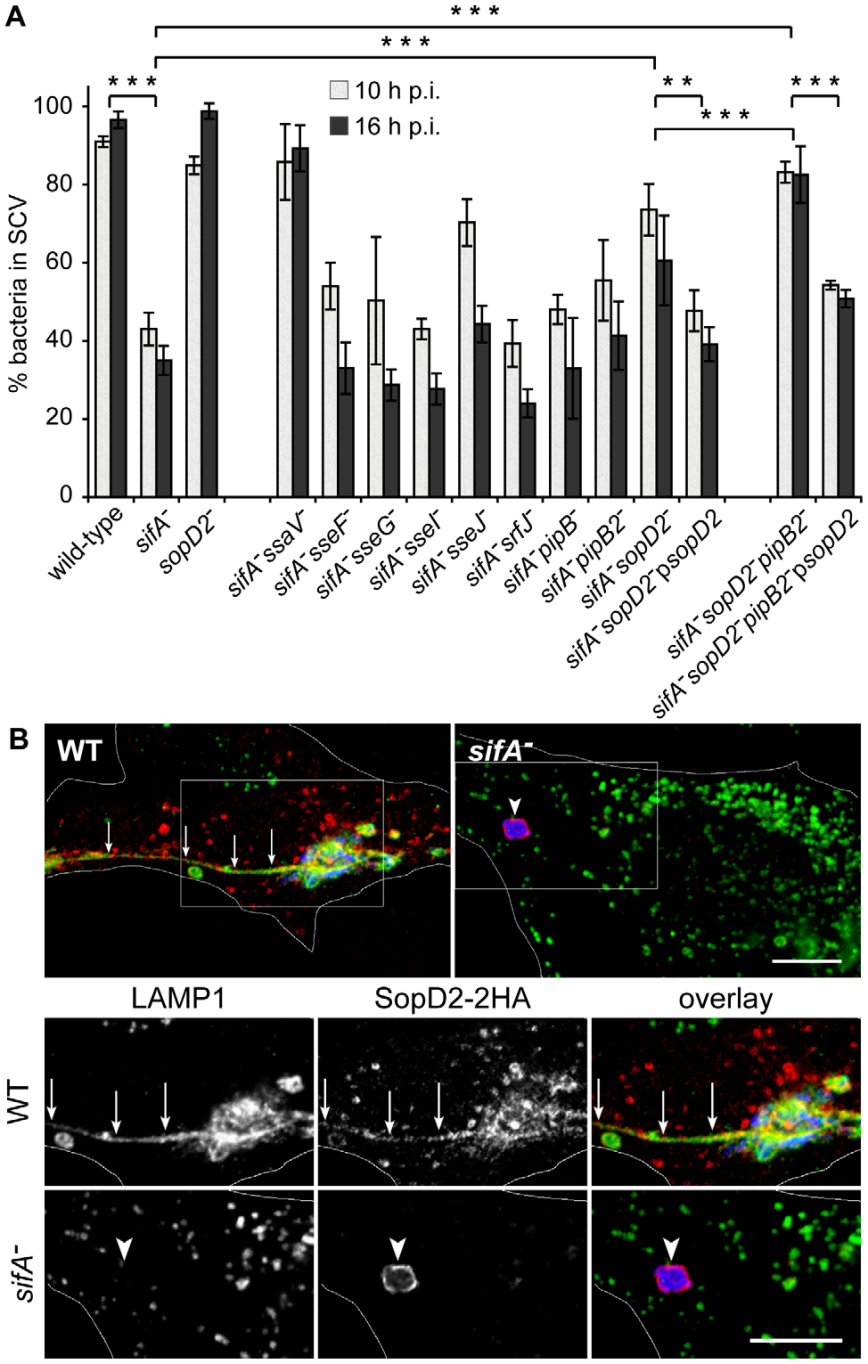
SopD2 localizes on and destabilizes *sifA* ^**−**^ SCVs. (A) HeLa cells were infected with various GFP-expressing *Salmonella* strains, fixed and immunostained for the SCV marker LAMP1. The percentages of bacteria in vacuoles were scored at 10 and 16 h p.i.. Values are means ± SD of 3 independent experiments. P-values: **, P<0.01; ***, P<0.001. (B) Cells were infected with wild-type (WT) or *sifA* ^−^ mutant strains expressing a 2HA-tagged version of SopD2 and imaged by confocal microscopy for GFP (blue), LAMP1 (green) and SopD2-2HA (red). Magnified insets showing single LAMP1 or SopD2-2HA labellings are presented in the lower panel. SopD2 localized on wild-type vacuoles and SIFs (arrows) but was solely present on SCVs in cells infected with a *sifA* ^−^ mutant (arrowhead). Scale bar, 10 µm.

A *sifA^−^* mutant is progressively released into the host cell cytoplasm whereas an *ssaV* ^−^ strain is enclosed in a stable vacuole [15]. This suggests that T3SS-2 effectors are responsible for the vacuole instability in the absence of SifA. We confirmed this hypothesis [15] using a *sifA^−^ssaV^−^* mutant whose vacuole stayed intact (Fig. 1A). In order to identify the T3SS-2 effector(s) involved in the instability of the *sifA^−^* SCV, we tested individually the effect of their deletion in a *sifA^−^* mutant strain. HeLa cells were infected with wild-type or mutant bacteria and the percentage of bacteria enclosed in LAMP1-positive vacuoles was determined. Most of the mutants behaved similarly to a *sifA^−^* strain in terms of SCV stability (Fig. 1A). In agreement with previous reports [23], [24], a *sifA* ^−^ *sseJ* ^−^ SCV was more stable than a *sifA* ^−^ SCV. Remarkably, the deletion of *sopD2* in a *sifA^−^* strain also significantly increased the percentage of bacteria present in SCVs. We noticed a modest but constant effect on SCV stability upon deletion of *pipB2* and found a more pronounced effect upon deletion of *sopD2* in a *sifA^−^pipB2^−^* strain (Fig. 1A). The complementation of *sifA^−^sopD2^−^* and *sifA^−^sopD2^−^pipB2^−^* strains with a plasmid for the expression of SopD2 reduced the percentage of bacteria in SCV (Fig. 1A). We observed a similar complementation using a 2HA-tagged version of SopD2 (*data not shown*), indicating that both native and 2HA-tagged proteins were functional. We localized SopD2 in infected HeLa cells. We found SopD2 on SCVs, SIFs and cytoplasmic vesicles in cells infected with wild-type *Salmonella* but its localisation was exclusively restricted to the SCV in absence of SifA (Fig. 1B). These results indicate that SopD2 is present on the *sifA^−^* SCV and that it plays a role in the membrane instability of this bacterial compartment.

**Figure 2:**
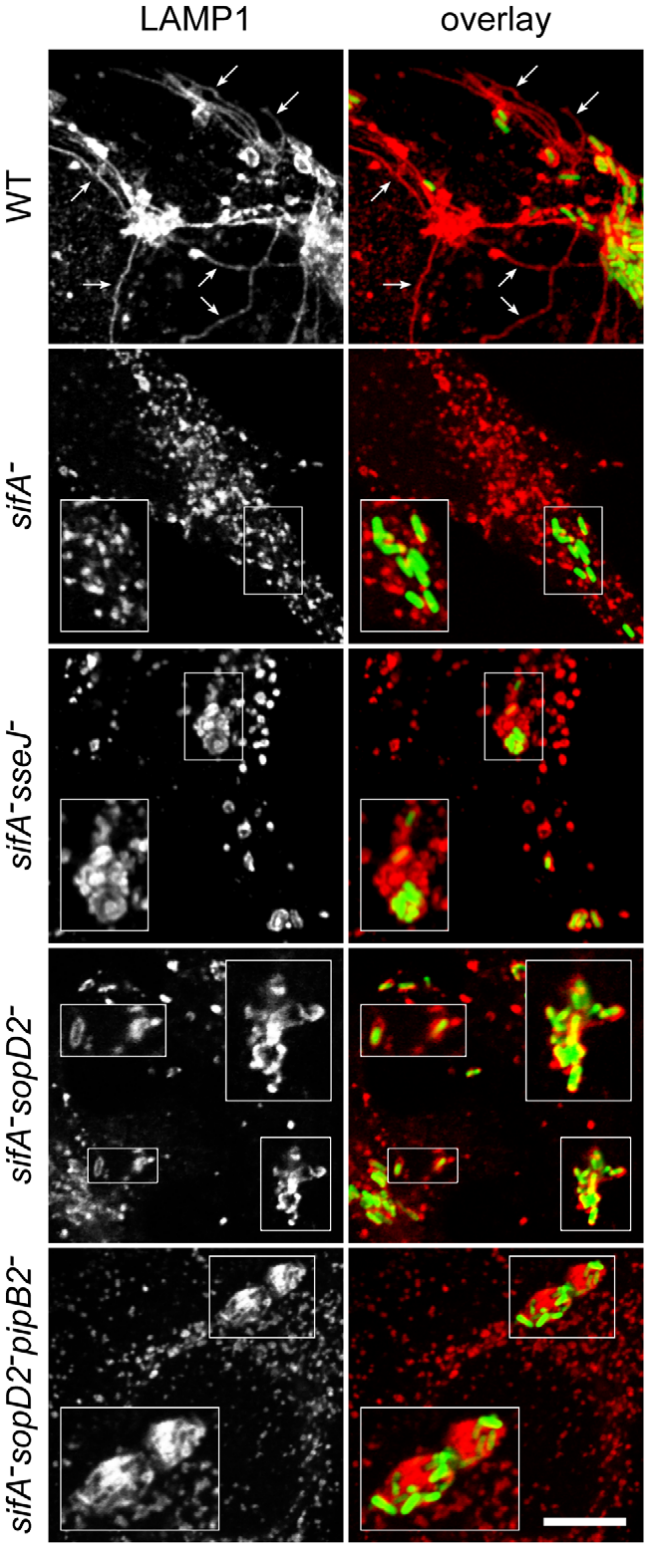
Immunofluorescence imaging of vacuoles enclosing various *Salmonella* strains. HeLa cells infected for 16 hours with GFP-expressing *Salmonella* strains were immunostained for the SCV marker LAMP1 and imaged by confocal microscopy for GFP (green) and LAMP1 (red). Insets illustrate the absence of vacuole enclosing *sifA* ^−^ bacteria and the shapes of *sifA^−^sseJ^−^, sifA^−^sopD2^−^* and *sifA^−^sopD2^−^pipB2^−^* vacuoles magnified twice. SIFs (arrows) were only seen in wild-type-infected cells. Scale bar, 10 µm or 5 µm for insets.

**Figure 3:**
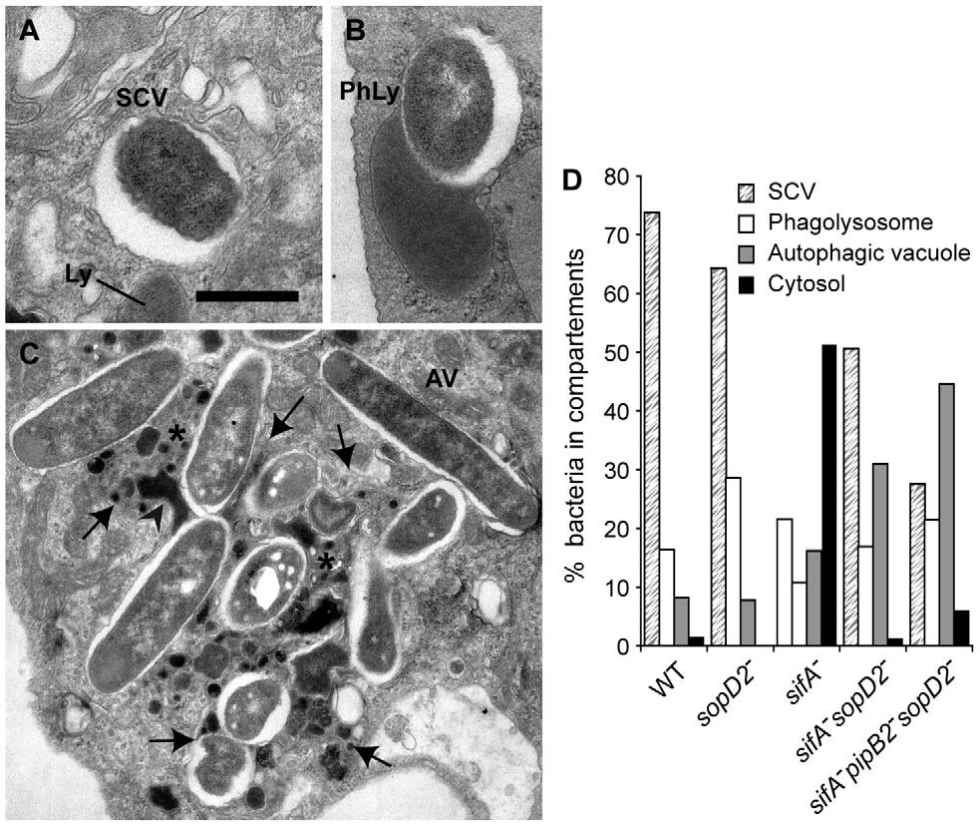
Morphological appearance of *Salmonella* vacuoles. BMDMs were infected with various *Salmonella* strains. At 12 h p.i., cells were fixed and processed for characterization of the vacuoles. (A–C) Morphological appearance of the various *sifA^−^sopD2^−^pipB2^−^* vacuoles. (A) View of a normal SCV. Lysosomes (Ly) do not fuse with this compartment. (B) A lysosome (Ly) is seen fusing with an SCV to form a phagolysosome (PhLy). In this PhLy, the bacterium is still morphologically intact. (C) View of an autophagic vacuole (AV). Cytoplasmic material (star) and bacteria can be seen in the AV, which also contains dense lysosomal material (arrowhead). Arrows indicate the membrane of the AV. Scale bar: 0.5 µm (A and B), 1,5 µm (C). (D) The fraction of bacteria enclosed in the different types of compartments as shown in A, B and C or in the cytosol was scored.

Confocal microscopic observations of infected HeLa cells revealed that wild-type SCVs contained a single bacterium while the *sifA^−^sseJ^−^* mutant formed micro-colonies of 2 to 6 bacteria (Fig. 2). The *sifA^−^sopD2^−^* mutant was present either in wild-type-like SCVs or in large vacuoles containing several bacteria and LAMP1 inclusions. *sifA^−^sopD2^−^pipB2^−^* vacuoles containing a single bacterium were rarely found and rather contained several bacteria with large inclusions of LAMP1 (Fig. 2). An electron microscopy (EM) qualitative and quantitative analysis was undertaken to better characterize both the vacuoles and the ultrastructural appearance of the bacteria within these compartments. Bone marrow-derived mouse macrophages (BMDMs) infected with the different *Salmonella* strains were fixed and processed for EM. Bacteria were observed in three types of compartments, namely normal SCVs (Fig. 3A), phagolysosomes (Fig. 3B) and autophagic vacuoles (Fig. 3C). The diverse types of vacuole were indistinguishable between strains but the percentages of bacteria within these different compartments or in the cytosol were different as shown by the scores at 12 hr post-invasion (p.i.) (Fig. 3D). As expected, over 75% of the WT and 60% of the *sopD2^−^* bacteria were confined to SCVs that usually contained a single bacterium, while at least 50% of the *sifA^−^* mutant had escaped the SCV. Deletion of *sopD2* in a *sifA^−^ or sifA^−^pipB2^−^* strain considerably reduced the number of cytosolic bacteria. However, while about 50% of the *sifA^−^sopD2^−^* bacteria resided in normal SCVs, the *sifA^−^sopD2^−^pipB2^−^* mutant was predominantly found in autophagic vacuoles. The latter usually contained several bacteria that were mostly morphologically intact. Finally, and for all strains, about 15 to 30% of the bacteria were enclosed in phagolysosomes. Bacteria were either morphologically intact as in Figure 3B (30 to 70% depending on strains) or undergoing degradation. All in all, the EM observations of infected BMDM confirmed that the deletion of *sopD2* in a *sifA^−^* mutant stabilizes the SCV and showed that the additional deletion of *pipB2* increases significantly the proportion of vacuoles of autophagic nature.

### Characterization of the *sifA* ^−^ *sopD2* ^−^ and *sifA* ^−^ *sopD2* ^−^ *pipB2* ^−^ vacuoles

**Figure 4:**
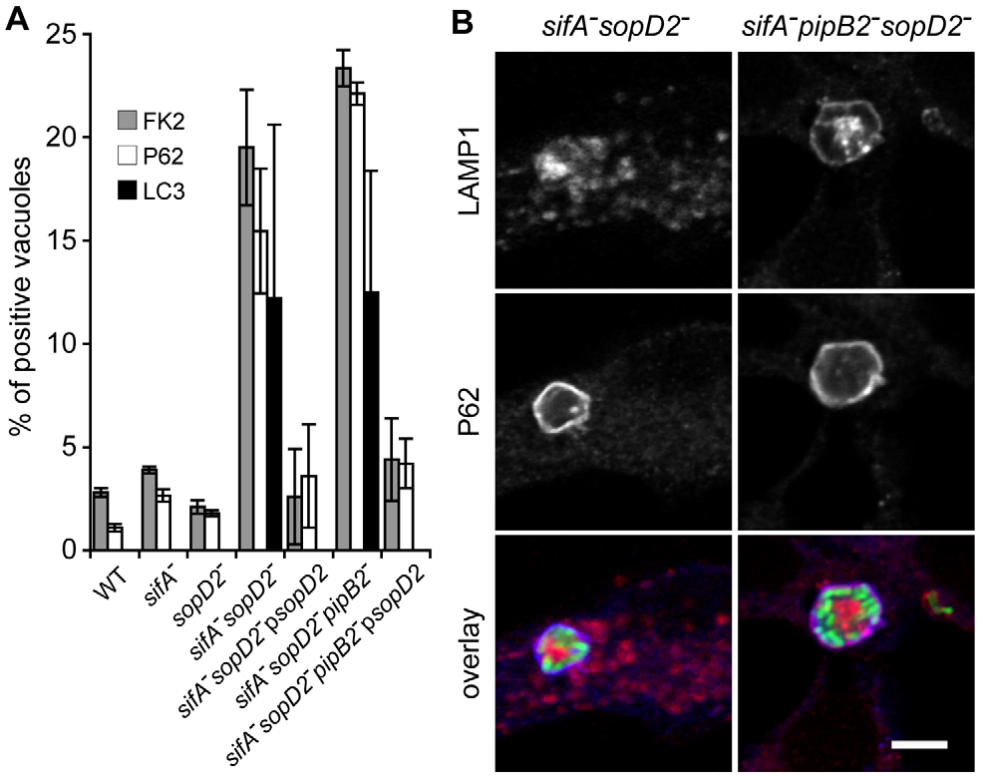
A fraction of *sifA* ^**−**^ *sopD2* ^**−**^ and *sifA* ^**−**^ *pipB2* ^**−**^ *sopD2* ^**−**^ vacuoles displays autophagic features. HeLa cells were infected for 16 h with GFP-expressing *Salmonella* strains, fixed and immunostained for FK2, P62 or LC3 and LAMP1 as a SCV membrane marker. (A) The percentages of FK2-, P62- or LC3- positive vacuoles were scored. Deletion of sopD2 increased the percentages of vacuoles positives for these markers. These phenotypes were complemented by expression of sopD2 from a plasmid. Values are means ± SD of 3 independent experiments. (B) HeLa cells were imaged by confocal microscopy for GFP (green), LAMP1 (red) and P62 (blue). Scale bar, 5 µm.

Since some of the mutants resided in autophagic vacuoles in BMDMs, we asked whether vacuoles previously observed in HeLa cells were also of an autophagic nature. To address this issue, we analysed by immunofluorescence the recruitment of different markers to the SCVs: i) the FK2 antibody that detects both mono- and poly-ubiquitinylated proteins, ii) P62, also known as sequestosome-1 (SQSTM1), that links ubiquitinated proteins to the autophagic machinery [25], [26], iii) LC3 that binds directly P62 and is a marker for membranes that are undergoing autophagy [27]. While little or no wild-type, *sifA* ^−^ or *sopD2^−^* SCVs were positive for these markers, around 20% of *sifA^−^sopD2^−^ or sifA^−^sopD2^−^pipB2^−^* SCVs were ubiquitinylated and positive for P62 and LC3 (Fig. 4A and B). The phenotypes displayed by *sifA^−^sopD2^−^* and *sifA^−^sopD2^−^pipB2^−^* mutants were complemented when a plasmid for the expression of SopD2 was introduced in the corresponding mutants (Fig. 4A).

To investigate whether *sifA^−^sopD2^−^* and *sifA^−^sopD2^−^pipB2^−^* mutants sequestered in the FK2/P62/LC3-positive vacuoles were viable or undergoing degradation, we analysed their metabolic activity using strains that expressed DsRed fluorescence under the control of an arabinose-inducible promoter. HeLa cells were infected and DsRed expression was induced at 14 h p.i. for 4 h before fixing the cells. All the bacteria within P62/LC3-positive vacuoles expressed DsRed (*data not shown*). Altogether, these results indicate that deletion of *sopD2* in *sifA^−^* or *sifA^−^pipB2^−^* mutants stabilizes the SCV membrane, which is in 20% of the cases positive for autophagic markers in HeLa cells. The bacteria contained in these vacuoles did not seem to be degraded, as they were still metabolically active at late times post-infection.

### Deletion of *sopD2* in a *sifA* ^−^ mutant restores several wild-type phenotypes

**Figure 5:**
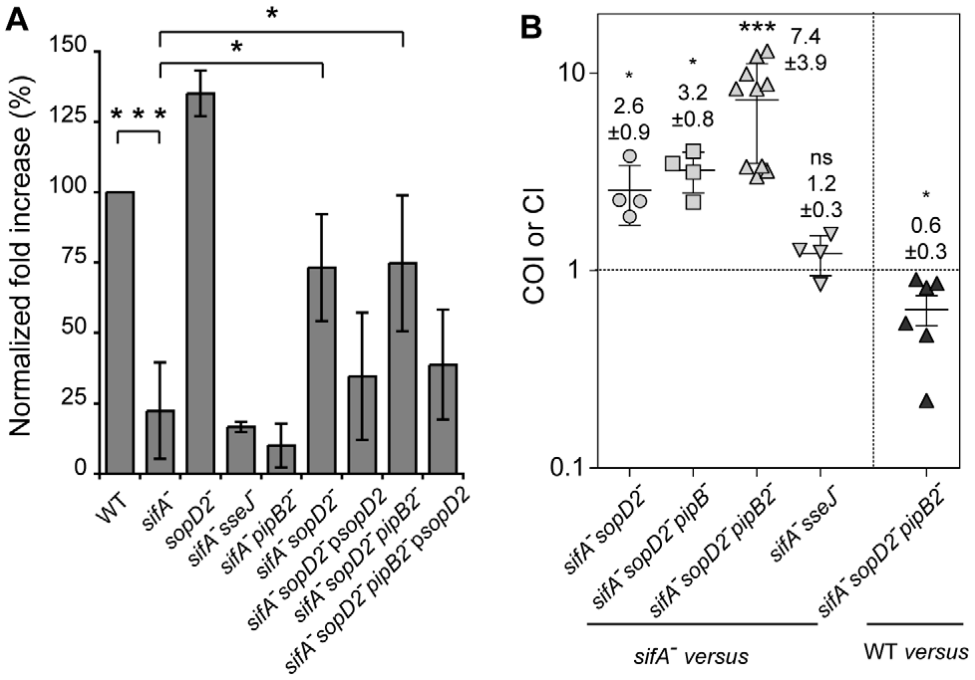
Deletion of *sopD2* promotes intra-macrophagic replication and virulenc*e* of *sifA* ^**−**^ and *sifA* ^**−**^ *pipB2* ^**−**^ mutant strains. (A) RAW264.7 macrophages were infected with wild-type and various mutant strains and lysed at 2 and 16 h p.i. for enumeration of intracellular bacteria. The values shown represent the fold increase calculated as a ratio of the intracellular bacteria between 16 and 2 h and normalized to that of the wild-type strain. Values are means ± SD (n = 6). (B) Both PipB2 and SopD2 impact the virulence attenuation of a *sifA* ^−^ mutant in the mouse model. Mice were inoculated i.p. with a mixture of two strains. Two days after infection, spleens were harvested for bacterial counts. COIs of *sifA^−^sopD2^−^* (n = 4), *sifA^−^sopD2^−^pipB^−^* (n = 4), *sifA^−^sopD2^−^pipB2^−^* (n = 10), *sifA^−^sseJ^−^* (n = 4), against the *sifA^−^* strain and the CI of *sifA^−^sopD2^−^pipB2^−^* against the wild-type (WT) strain (n = 6) were determined. Each symbol represents the COI/CI from one mouse, and horizontal bars correspond to the means ± SD. A one-sample *t* test was used to determine whether the mean value was significantly different from 1. (A and B) P-values: ns, not significant; *, P<0.05; ***, P<0.001.

Given the above data and since *Salmonella* is unable to replicate in the macrophage cytosol [16] we asked whether deletion of *sopD2* would restore bacterial replication in cultured macrophages. RAW 264.7 mouse macrophages were infected with wild-type or mutant bacteria and the fold increase of intracellular bacteria between 2 and 16 h after phagocytic uptake was determined (Fig. 5A). As expected [15], a *sifA* ^−^ mutant had a strong replication defect and a *sifA* ^−^ *sseJ* ^−^ mutant [24] did not replicate better than a *sifA* ^−^ mutant. In contradiction to former studies [28], a strain lacking *sopD2* was not impaired in intracellular replication (Fig. 5A). Deletion of *pipB2* in a *sifA* ^−^ mutant had no effect on bacterial replication (Fig. 5A) [14]. However, deletion of *sopD2* in both *sifA* ^−^ and *sifA* ^−^ *pipB2* ^−^ mutants promoted bacterial replication. Expression of SopD2 from a plasmid reduced replication of the *sifA^−^sopD2^−^* and *sifA^−^sopD2^−^pipB2^−^* mutant strains (Fig. 5A).

Next, we tested the virulence of these *Salmonella* mutants *in vivo* by performing mixed infections in C57BL/6 mice [29]. We found that *sifA* ^−^ and *sifA* ^−^ *sseJ* ^−^ mutant strains exhibited similar virulence (COI = 1.2±0.9) (Fig. 5B) while the *sifA^−^sopD2^−^* strain was slightly but significantly more virulent that the *sifA^−^* mutant (COI = 2.6±0.9) (Fig. 5B). Since deletion of *pipB2* increases moderately the virulence of a *sifA* ^−^ mutant [14] and since deletion of *sopD2* and *pipB2* had an additive effect on the vacuole stability (Fig. 1A), we also tested a *sifA^−^sopD2^−^pipB2^−^* mutant. The virulence of this mutant was highly increased (COI = 7.35±3.8). Deletion of *pipB* that encodes a PipB2 homolog did not significantly change the virulence of a *sifA^−^sopD2^−^* mutant. Compared to the wild-type strain, the *sifA^−^sopD2^−^pipB2^−^* mutant exhibited only a modest attenuation (CI of 0.63±0.27), while our previous data showed a CI of about 0.1 for a *sifA* ^−^ mutant [14]. These results indicate that the stabilisation of the *sifA* ^−^ *sseJ* ^−^ vacuole is not sufficient to increase the virulence. They also indicate that *pipB2* and *sopD2* contribute independently to the virulence attenuation of a *sifA^−^* mutant.

### SopD2 contributes to kinesin-1 retention on the *sifA* ^−^ vacuole

**Figure 6:**
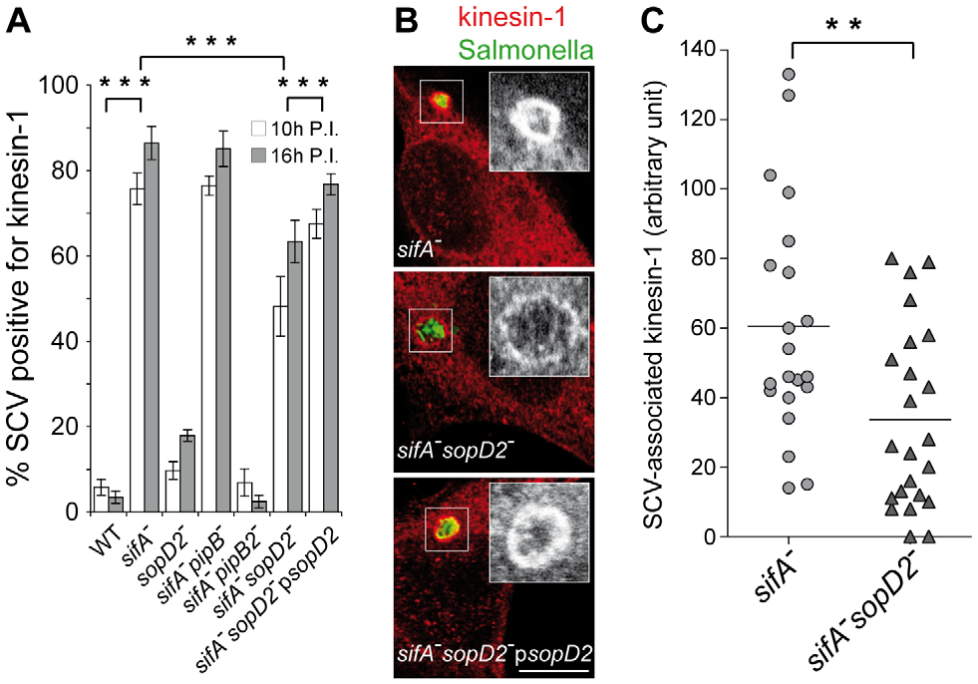
SopD2 influences the retention of kinesin-1 on the bacterial vacuole. HeLa cells were infected with various GFP-expressing strains fixed and immunostained for kinesin heavy chain and LAMP1 as a SCV membrane marker (not shown). (A) Deletion of *sopD2* reduces the percentage of kinesin-1-positive *sifA* ^−^ SCVs. Kinesin-1-positive SCVs were scored at 10 and 16 h p.i.. Values are means ± SD of 3 independent experiments. (B and C) Deletion of *sopD2* decreases the amount of kinesin-1 on *sifA^−^* SCVs. (B) HeLa cells infected for 16 h were imaged by confocal microscopy for GFP (green) and kinesin-1 (red). Scale bar, 10 µm. (C) Quantitative analysis of confocal images was used to determine the relative staining intensities for kinesin-1 of *sifA^−^* and *sifA^−^sopD2^−^* vacuoles. Each point corresponds to the analysis of one SCV. Results of one experiment are shown. This experiment was repeated three times, with comparable results. P-values: ** P<0.01; *** P<0.001.

PipB2 operates as a linker for kinesin-1 on the SCV [14] and the *sifA* ^−^ vacuole is characterized by a high accumulation of this microtubule motor [13]. In order to define whether SopD2 plays a role in this process, we scored the percentage of SCVs positive for kinesin-1 in infected HeLa cells. In agreement with former studies [13], [14], most of the *sifA* ^−^ SCVs accumulated kinesin and this recruitment was completely abrogated upon deletion of *pipB2* but not *pipB* (Fig. 6A). Deletion of *sopD2* in a *sifA* ^−^ mutant significantly decreased the percentage of kinesin-1-positive SCVs, although not to the same extent as the deletion of *pipB2*. This deficit was complemented when a plasmid for the expression of SopD2 was introduced in the *sifA* ^−^ *sopD2* ^−^ mutant, thus confirming the specificity of this observation. We also observed and quantified that deletion of *sopD2* in a *sifA* ^−^ mutant reduced the vacuolar membrane load in kinesin-1 (Fig. 6B and C). These findings show that SopD2 increases both the percentage of vacuoles positive for kinesin-1 and the amount of kinesin-1 present on *sifA* ^−^ vacuoles.

### SopD2 inhibits the peripheral transport of kinesin-1-positive vesicles

**Figure 7:**
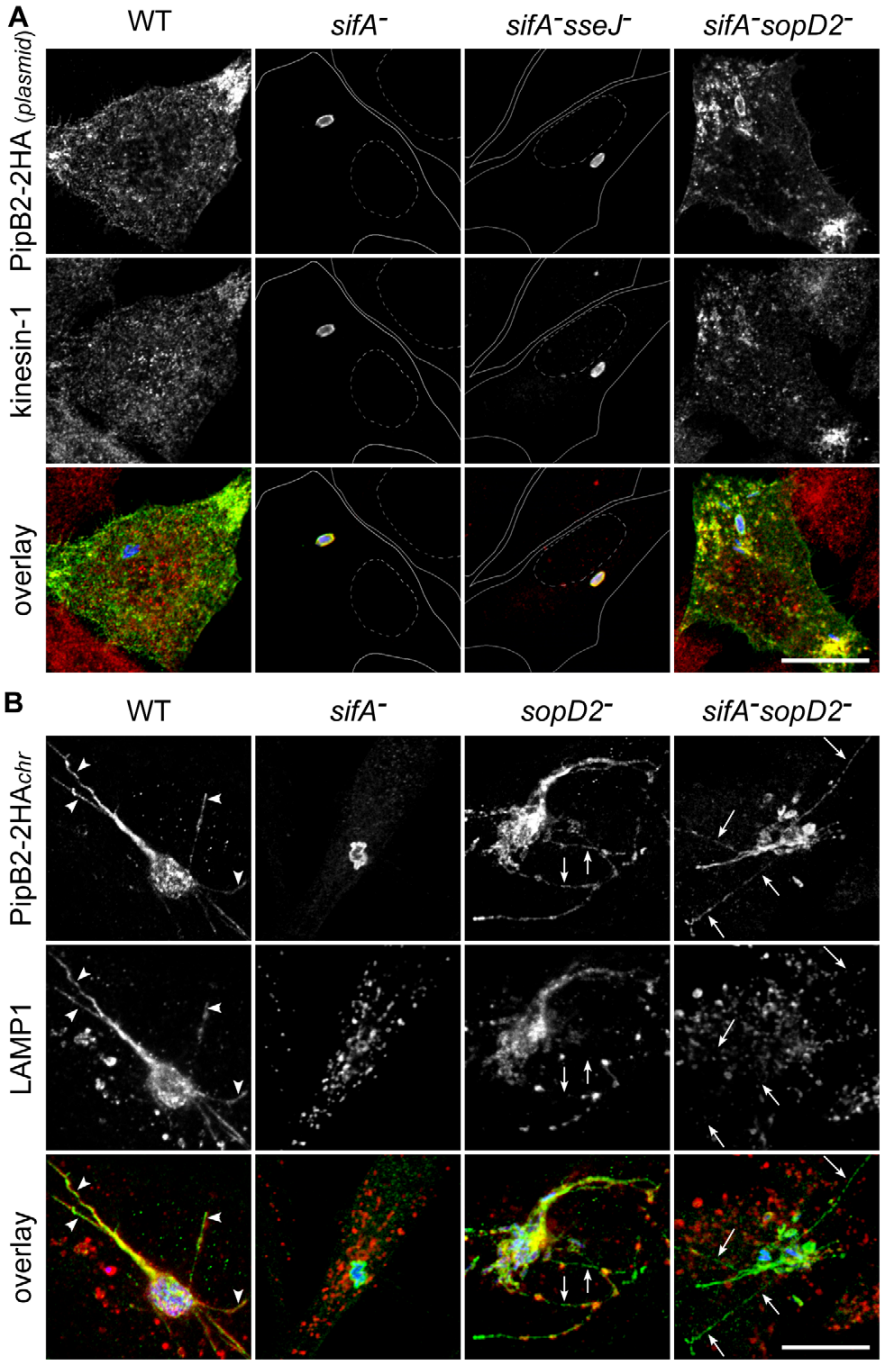
SopD2 inhibits the formation of effector-positive vesicles and tubules. (A) SopD2 blocks the formation of PipB2- and kinesin-1-positive vesicles. HeLa cells were infected for 16 hours with strains over-expressing PipB2-2HA from a plasmid. Fixed cells were immunostained and imaged for HA (green), kinesin heavy chain (red) and GFP (blue). Solid and dotted lines (central rows) delineate cells and nuclei, respectively. PipB2- and kinesin-positive vesicles accumulate at the periphery of cells infected with wild-type and *sifA* ^−^ *sopD2* ^−^ strains while these molecules accumulated on *sifA* ^−^ and *sifA* ^−^ *sseJ* ^−^ SCVs. (B) SopD2 negatively regulates the formation of PipB2-positive but LAMP1-negative tubules. HeLa cells were infected with strains (blue) expressing 2HA-tagged PipB2 from the chromosome. Fixed cells were immunostained and imaged for HA (green), LAMP1 (red) and GFP (blue). PipB2- and LAMP1-positive tubule (arrowheads) and PipB2-postive but LAMP1-negative tubule (arrows) are indicated. (A and B) Scale bar, 20 µm.

Since we observed removal of a significant amount of kinesin-1 upon deletion of *sopD2* in a *sifA* ^−^ mutant, we asked whether it could result from the restoration of a vesicular transport from the SCV. To address this point, we infected HeLa cells with *Salmonella* strains expressing 2HA-tagged PipB2 from a plasmid. Consistent with the previous studies [20], [22], we observed the presence of PipB2/kinesin-1-positive vesicles at the periphery of cells infected with wild-type *Salmonella*, while these molecules accumulated on *sifA* ^−^ SCVs and also on *sifA* ^−^ *sseJ* ^−^ SCVs (Fig. 7A). Strikingly, we observed vesicles positive for both kinesin-1 and PipB2 at the periphery of HeLa cells (Fig. 7A) and RAW 264.7 macrophages (data not shown) infected with a *sifA* ^−^ *sopD2^−^* mutant, thus giving images very similar to cells infected with wild-type *Salmonella*. These results clearly indicate that, in contrast to SseJ, SopD2 inhibits the formation and/or transport of PipB2/kinesin-1-positive vesicles from *sifA* ^−^ SCVs.

### SopD2 regulates the formation of T3SS-2 effector-positive tubules

To gain better insights into the genesis of PipB2/kinesin-1-positive vesicles, and to avoid the over-expression of PipB2-2HA, we engineered *Salmonella* strains expressing PipB2-2HA from the chromosome. We observed that in HeLa cells PipB2 localized on *sifA* ^−^ SCVs and, very surprisingly, on the *sifA* ^−^ *sopD2^−^* SCVs and tubular structures that extended from the vacuole (Fig. 7B). These tubular structures are reminiscent of SIFs. Therefore we examined whether SIFs, which are LAMP1-positive tubular structures [18] and which have never been observed in the absence of SifA, were formed in concomitant absence of SifA and SopD2. Most tubular structures were positive for both LAMP1 and PipB2 in cells infected with wild-type *Salmonella*, while tubules formed in cells infected with the *sifA^−^sopD2^−^* mutant were negative for LAMP1 (Fig. 7B). In agreement with a previous report [30], a discontinuous distribution of LAMP1 along effector-positive tubule and also LAMP1-negative tubules (Fig 7B) were observed in the sole absence of SopD2.

**Figure 8:**
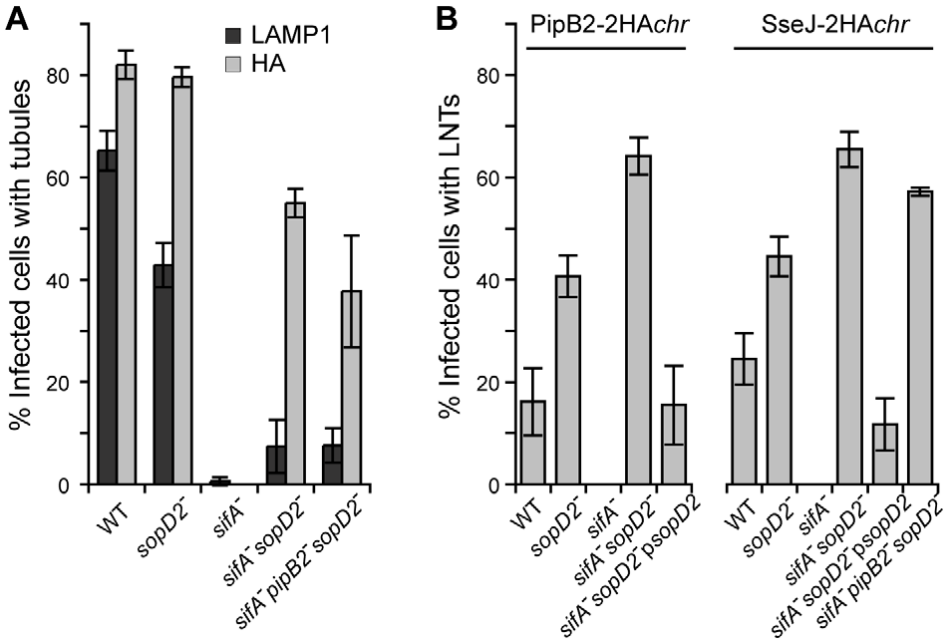
Scoring of SIFs and effector-positive tubules in infected HeLa cells. HeLa cells were infected for 16 h with strains expressing SseJ-2HA or PipB2-2HA from the chromosome. (A) Cells were immunolabelled for LAMP1 and SseJ-2HA. The percentage of infected cells containing LAMP1- or HA-positive tubules was scored. (B) Cells were immunolabelled for LAMP1 and HA. The percentage of infected cells containing LAMP1-Negative Tubules (LNTs) was scored. (A and B) Values are means ± SD of 3 independent experiments.

Strains expressing SseJ-2HA from the chromosome were used to score the occurrence of these membranous tubular structures. SIFs (LAMP1-positive tubules) were present in nearly 65 and 45% of cells infected with wild-type and *sopD2* ^−^ strains, respectively, while SseJ-positive tubules were seen in about 80% of cells infected with either strain (Fig. 8A). In agreement with previous data [17], [31], neither SIFs nor effector-positive tubules were detected in cells infected with a *sifA^−^* mutant. SseJ-positive tubules were formed in 55% of cells infected with *sifA^−^sopD2^−^* mutant, while SIFs were barely detected. A *sifA^−^sopD2^−^* strain is therefore able to form T3SS-2 effector-positive and LAMP1-negative structures that are hereafter referred to as LNT for LAMP1-Negative Tubule. We scored LNTs in cells infected with strains expressing either 2HA-tagged PipB2 or SseJ from the chromosome and very similar results were obtained (Fig. 8B). LNTs were more frequently found in cells infected with a *sopD2* ^−^ mutant than with wild-type *Salmonella*. Since PipB2 is involved in the centrifugal extension of SIFs [32], we tested whether this effector was involved in the formation of LNTs. A *sifA* ^−^ *sopD2* ^−^ *pipB2* ^−^ strain was still able to form effector-positive tubules (Fig. 6C and D). However these tubules were much shorter compared to those of the *sifA^−^sopD2^−^* mutant (*data not shown*), indicating that PipB2 participates to their elongation. Conversely, the accumulation of peripheral vesicles in cells infected with *Salmonella* over-expressing PipB2 (Fig. 7A) suggests that these vesicles come from the PipB2-mediated fragmentation of LNTs. All in all, these results indicate that SopD2 inhibits the formation of LNTs and that SopD2 and SifA have antagonistic roles regarding their formation, while PipB2 is involved in their centrifugal extension.

### Characterisation of LNTs

**Figure 9:**
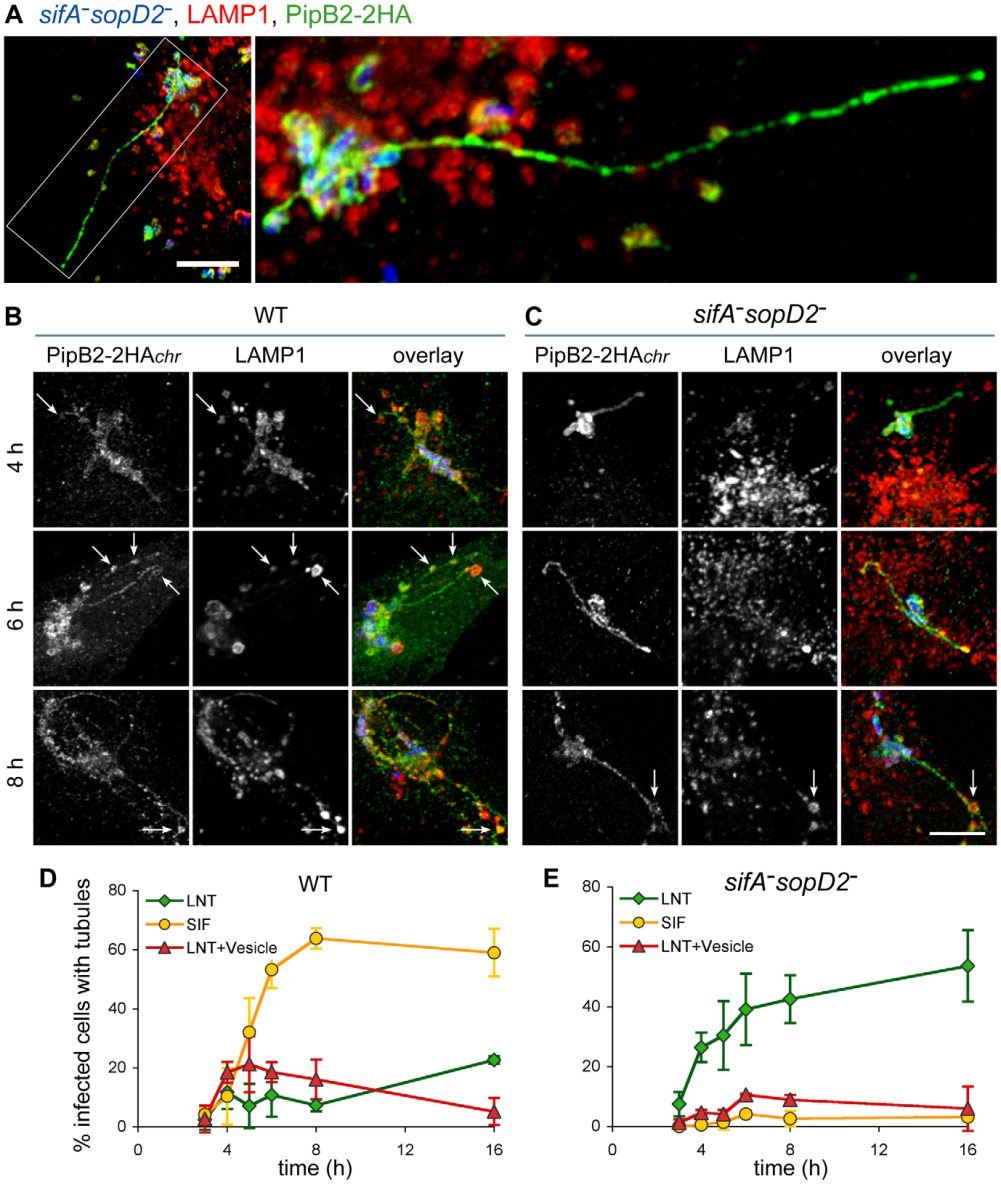
Structure and kinetics of formation of LNTs. HeLa cells were infected with GFP-expressing wild-type or *sifA^−^sopD2* strains (blue) and immunostained for LAMP1 (red) and PipB2-2HA (green) expressed from the chromosome. Images in (A) present a cell infected for 16h with the *sifA^−^sopD2^−^* mutant strain. The right image is the delineated zone magnified 3 times. LNTs as long as 30–40 µm were observed. Scale bar, 10 µm (left image) and 3,3 µm (right image). (B to C) Cells were fixed at 4, 6 and 8 h, and imaged. Short LNTs were observed as early 4 h p.i.. LNTs contacting LAMP1-positive vesicles were frequently observed (arrows in B and C). Scale bar, 10 µm. (D and E) LNTs, LNTs contacting LAMP1-positive vesicles and SIFs were scored at different time-points. Values are mean ± SD of 3 independent experiments.

To gain more insights into the structure and the kinetics of LNT formation, we observed and scored both LNTs and SIFs at selected times post-infection. At 16 p.i. LNTs appeared as continuous tubular structures emerging from SCVs and extending towards the cell periphery. PipB2 localized all along LNTs but presented a patch-like distribution (Fig. 9A). As we noticed that LNTs were frequently in contact with LAMP1-positive vesicles, both in wild-type and *sifA* ^−^ *sopD2* ^−^ infected cells (arrows in Fig. 9B and C), these structures (LNT in contact with LAMP1 vesicle) were scored separately. In wild-type infected cells, the three types of structures appeared simultaneously at 3–4 hours p.i.. Then, the occurrence of SIFs increased and reached a plateau at 8 hours p.i., while the percentage of cells with LNTs and LNTs in contact with LAMP1-vesicles remained lower than 20% of infected cells starting from 4 hours p.i. (Fig. 9B and D). The *sifA^−^sopD2^−^* strain predominantly generated LNTs. The percentage of LNT contacting LAMP1-vesicles was reduced compared to cells infected with the wild-type strain, especially at early times of infection (Fig. 9C and E).

**Figure 10:**
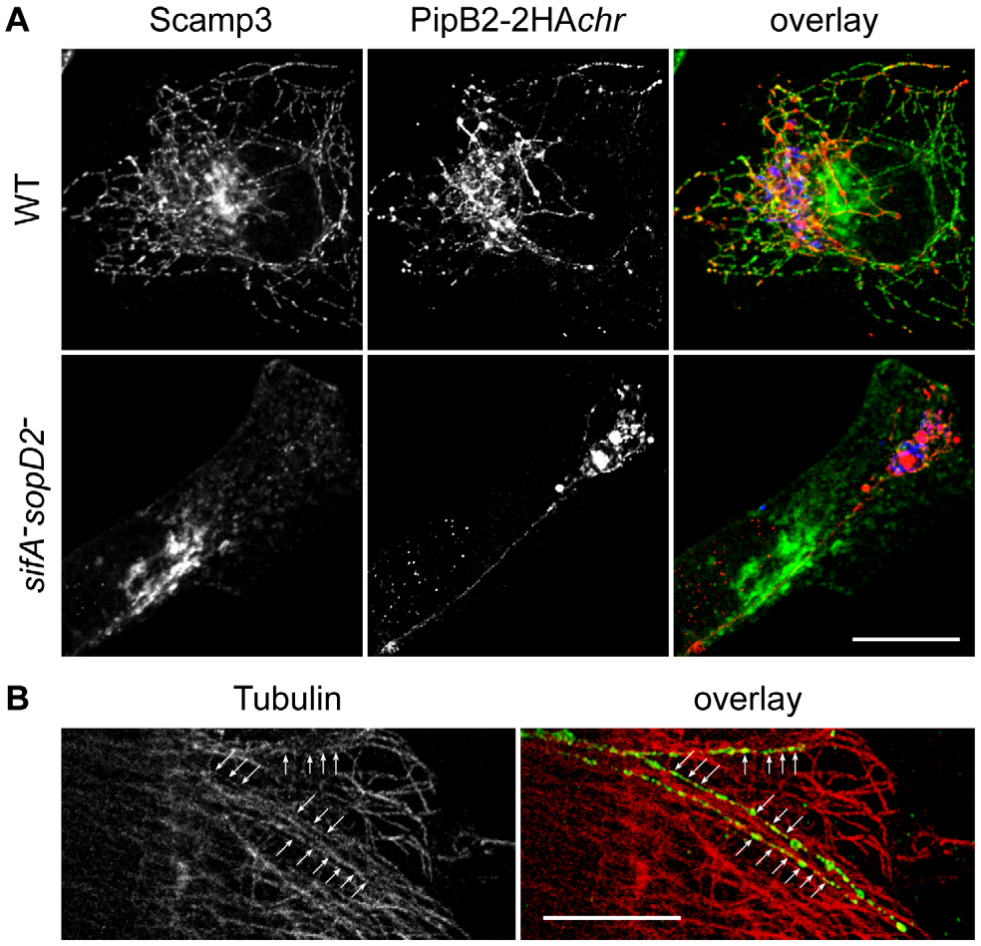
Characterization of LNTs. (A) LNTs are negative for Scamp3. Infected HeLa cells were fixed at 16 h p.i., immunolabelled and imaged for GFP (blue), PipB2-2HA (red) expressed from the chromosome and Scamp3 (green). Scale bar, 20 µm. (B) LNTs (green) are aligned (arrows) along microtubules (red). Scale bar, 10 µm.

In addition to SIFs, *Salmonella* induces the formation of SISTs, which are Scamp3-positive tubules that lack late endosomal/lysosomal proteins [19]. Therefore, we asked whether LNTs correspond to SISTs. In wild-type infected cells, Scamp3 was found on tubules that were frequently positive for PipB2. Conversely, Scamp3 was distributed in the perinuclear area both in non-infected (*data not shown*) and cells infected with the *sifA^−^sopD2^−^* mutant (Fig. 10A). These results suggest that LNTs form independently from SISTs.

Finally, we examined LNTs for the presence of other markers and analyzed their sensitivity to several treatments (Table 1). Unlike SIFs, LNTs were negative for Rab7, the lysosomal glycoproteins LAMP1, CD63 and the late endosomal lipid LBPA. Similar to SIFs, LNTs were positive for the vacuolar ATPase (vATP) and contained cholesterol. As listed in Table 1, LNTs were negative for most endocytic and exocytic markers. The host protein SKIP was not involved in LNT formation. LNTs were found aligned with microtubules (Fig. 10B) and their extension and maintenance were dependent on an intact microtubule network. Treatment with nocodazole at 3h p.i., when LNTs start to form, impaired their formation. Integrity of the LNT network was affected by disruption of microtubules at 14h p.i.. Treatment with the actin-disrupting agent cytochalasin D had any impact on LNT stability or formation. In contrast to SIST, brefeldin A treatment of infected cells did not significantly affect LNT formation (Table 1), suggesting that a functional exocytic pathway is not required. These data confirm that LNTs and SISTs are distinct structures. They also indicate that LNTs are likely to form along microtubules, but are devoid of most markers associated with SIFs. Together, these results demonstrate the presence of at least three types of tubules in *Salmonella* infected cells: SIFs [18], SISTs [19], and LNTs.

**Table 1:** Features of LNT, SIF and SIST.

Marker (compartment)*	SIFs	SISTs	LNTs	References
LAMP1 (LE/Lys)	+	−	−	[18]
Scamp3	+/−	+	−	[19]
T3SS-2 effector	+	+	+	[15], [17], [19], [23], [28], [32], [40], [41], [42]
EEA1	−	n.d.	−	[43]
Rab5 (EE)	−	n.d.	−	[33]
Rab7 (LE)	+	n.d.	−	[33]
Rab9 (LE)	−	n.d.	−	
vATPase (LE/Lys)	+	−	+	[15]
Yip3 (LE)	−	n.d.	−	
Syntaxin 7 (LE)	n.d.	n.d.	−	
LBPA (LE)	+	n.d.	−	[33]
CD63 (LE/Lys)	+	−	−	[15]
Mannose 6-Phosphate Receptor (LE, TGN)	−	n.d.	−	[44], [45]
VPS26 (TGN)	−	n.d.	−	
Syntaxin 6 (TGN)	−	n.d.	−	
P230 (TGN)	−	n.d.	−	
GM 130 (*cis* Golgi)	−	n.d.	−	
Calreticulin (ER)	−	n.d.	−	
Calnexin (ER)	−	n.d.	−	
Dynein (P150^glued^)	−	n.d.	−	[13]
Cholesterol	+	n.d.	+	[33]
**Susceptibility**				
Nocodazole	+	+	+	[19], [35], [46]
Cytochalasin D	−	n.d.	−	[35], [46]
Brefeldin A	−	+	−	[19], [33]
SKIP RNAi	+	n.d.	−	[13]

EE, early endosome; ER, endoplasmic reticulum; LE, late endosome; LE/Lys, late endosome/lysosome; TGN, *trans*-Golgi network.

n.d., not determined.

## Discussion

In this work, we demonstrate that the T3SS-2 effector SopD2 is present on the *sifA* ^−^ SCV and that it contributes to its membrane instability. *sifA^−^sopD2^−^* and *sifA^−^pipB2^−^sopD2^−^* mutant strains are enclosed and replicate in steady vacuoles. As previously shown [23], [24], a *sifA* ^−^ *sseJ* ^−^ mutant resides also in a stable vacuole, which, however, does not support bacterial replication. In addition, in contrast to *sifA^−^sopD2^−^*, a *sifA* ^−^ *sseJ* ^−^ strain is not more virulent than a *sifA* ^−^ mutant. Therefore, it appears that a stable vacuole is necessary but not sufficient for intracellular replication and virulence in the mouse model.

In both epithelial cells and macrophages, a fraction of *sifA^−^sopD2^−^* and *sifA^−^pipB2^−^sopD2^−^* mutant bacteria resides in autophagosome-like vacuoles in which they remain metabolically active. This indicates that the harmonized activities of SifA, SopD2 and PipB2 are necessary for the formation and/or maintenance of a canonical SCV. It also shows that *Salmonella* is capable of surviving within compartments derived from the autophagic pathway. Yet, it remains unclear why a fraction of SCVs gets ubiquitinylated and recognized by the autophagy machinery. One possibility is that SopD2 is somehow involved in the suppression of autophagy, thereby protecting *Salmonella* from xenophagy. However, this hypothesis is inconsistent with the fact that this phenotype was not observed in the sole absence of SopD2.

SifA and SopD2 have antagonistic activities regarding the stability of the bacterial vacuole, the intra-macrophagic replication and the virulence. However, the deletion of *sopD2* in a *sifA* ^−^ background is not sufficient to recover certain wild-type phenotypes. Noticeably, a *sifA^−^sopD2^−^* mutant is not able to trigger the formation of SIFs and SISTs, pointing out to a crucial role for SifA in the formation of these membranous tubular structures. Also, a *sifA^−^sopD2^−^* mutant still accumulates kinesin-1, but to a lower extent than a *sifA^−^* mutant. This subtle but reproducible change results from the increased SCV membrane exchange activity, which in HeLa cells and macrophages infected with a *sifA^−^sopD2^−^* strain expressing PipB2-2HA from a plasmid, leads to the accumulation of effector- and kinesin-positive vesicles at the cell periphery. In cells infected with bacteria expressing physiological levels of this effector, this leads to the formation of tubules, the LNTs, which extend from the bacterial vacuole. Our data indicate that SopD2 inhibits the formation of LNT. Consistently cells infected with a *sopD2^−^* mutant exhibit more effector-positive tubules, which were either negative for LAMP-1 or presented a patchy distribution of this marker. These tubules have been previously described and named “pseudo-SIFs” [30]. This observation possibly reflects the activity of SopD2, which, even in the presence of SifA, restricts the formation of effector-positive tubules.

Because LNTs do not require SifA nor an intact secretory pathway for their formation, these tubules are undoubtedly distinct from SIFs and SISTs. It is unknown which intracellular compartment LNTs derive from. While SISTs appear to originate from the secretory pathway, SIFs are composed of both endosomal and TGN-derived material [18], [19], [33]. LNTs were found to be positive for the vATPase but were devoid of all other exocytic and endocytic markers tested so far. Yet, LNTs are, like SCVs, enriched in T3SS-2 effectors indicating that they might extend from the SCV membrane. Strikingly, effector-positive tubules projecting from the SCV that do not localize with either endocytic or Golgi markers were recently identified by live-cell imaging [34]. Also, how LNTs are generated remains unknown. Like SIFs and SISTs [18], [19], [32], [35], their formation is dependent on an intact microtubule network. We observed that LNTs were shorter in absence of PipB2, indicating a role for kinesin-1 in the extension of LNTs along microtubules. Indeed, the accumulation of vesicles observed in cells infected with a *sifA^−^sopD2^−^* mutant that over-expresses PipB2 from a plasmid likely reflects a kinesin-mediated fragmentation of LNT into vesicles.

Interestingly, the presence of LNT was concomitant with an increased stability of the SCV membrane. This suggests that LNTs might help recruit and transport membrane towards SCVs. Microscopic observations revealed that LAMP1-positive vesicles were occasionally enclosed in T3SS-2 effector-positive tubules. Indeed, the LNTs seem to wrap around LAMP1 vesicles. The close apposition between LNTs and vesicles might favour fusion and transfer of membrane from these vesicles to LNTs and subsequently to SCVs. This recruitment would ensure a supply in membrane and promote the stability of the vacuole. We propose that, in the absence of SifA and SopD2, close apposition between LNTs and vesicles is not efficient enough to trigger a massive recruitment of lysosomal membrane markers to the SCV and tubules but that these contacts generate a retrograde flow of membrane sufficient to stabilize the bacterial vacuole. LNTs were not observed in cells infected with a *sifA^−^sseJ^−^* mutant and sseJ-2HA was even used as marker for LNT. Thus, the instability of the *sifA* ^−^ vacuole results from both the enzymatic activity of SseJ [23], [24] and the inhibition of membrane exchange activity of SopD2.

Altogether this work has identified SopD2 as a major regulator of SCV stability and as an inhibitor of LNTs formation. LNTs are a novel kind of tubules that may represent precursors of SIFs and SISTs. The formation of LNTs likely involves additional T3SS-2 effectors and our work opens the way for their identification and characterization. Another interesting challenge will be to understand the cell biology of LNT initiation and the molecular mechanism by which SopD2 blocks LNT formation.

## Materials and Methods

### Ethics statement

Animal experimentation was conducted in strict accordance with good animal practice as defined by the French animal welfare bodies (Law 87–848 dated 19 October 1987 modified by Decree 2001-464 and Decree 2001-131 relative to European Convention, EEC Directive 86/609). All animal work was approved by the Direction Départmentale des Services Vétérinaires des Bouches du Rhônes (authorization number 13.118 to S.M.).

### Bacterial strains, plasmids, and culture conditions

The *S. enterica* serovar Typhimurium strains and plasmids used in this study and their relevant characteristics are listed in Tables 2 and 3. Strains were cultured in LB broth (Difco) at 37°C. Ampicillin (50 µg/ml), kanamycin (50 µg/ml), and chloramphenicol (50 µg/ml) were added when required.

**Table 2:** *Salmonella* strains used in this study.

Name	Parental Strain	Characteristics	Source
12023	12023	wild-type	[47]
DH215sc4	12023	*sifA^−^*	[14]
ΔsopD2	12023	*sopD2*::cm	Junkal Garmendia and David Holden
ΔsopD2	12023	*sopD2*::kan	Junkal Garmendia and David Holden
TH120	12023	*sifA*::kan, *sseF* ^−^	[14]
TH121	12023	*sifA* ^−^, *srfJ*::pGF704	[14]
TH122	12023	*sifA* ^−^, *sopD2*::kan	this study
TH123	12023	*sifA* ^−^, *sseG*::kan	[14]
TH125	12023	*sifA* ^−^, *ssaV^−^*::aphT	[14]
TH127	12023	*sifA* ^−^,*sseI*::pGF704	[14]
TH129sc4	12023	*sifA* ^−^, *sseJ* ^−^	[14]
TH145	12023	*sifA* ^−^, *pipB*::kan	[14]
TH146sc4	12023	*sifA* ^−^, *pipB2* ^−^	[14]
TH150	12023	*sifA* ^−^, *sopD2*::cm	[14]
TH153	12023	*sifA* ^−^, *sopD2*::cm, *pipB*::kan	this study
TH154sc	12023	*sifA* ^−^, *sopD2* ^−^, *pipB*2^−^	this study
NS021	12023	*sopD2*::kan,p*sopD2-*2HA	this study
NS022	12023	*sifA* ^−^, *sopD2* ^−^, *pipB*2^−^, p*sopD2-*2HA	this study
NS023	12023	*sifA* ^−^, *sopD2*::kan, p*sopD2-*2HA	this study
SseJ-2HAchr	12023	*sseJ-*2HAchr (chromosomal)	[48]
AAG006	12023	*sifA* ^−^, *sseJ-*2HAchr (chromosomal)	this study
AAG008	12023	*sopD2* ^−^, *sseJ-*2HAchr	this study
AAG013	12023	*sifA* ^−^, *sopD2* ^−^, *sseJ-*2HAchr	this study
AAG017	12023	*sifA* ^−^, *sopD2* ^−^, *pipB2* ^−^,*sseJ-*2HAchr	this study
AAG020	12023	*pipB2-*2HAchr	this study
AAG021	12023	*sopD2-*2HAchr	this study
AAG022sc4	12023	*sifA* ^−^, *pipB2-*2HAchr	this study
AAG023scs	12023	*sopD2* ^−^, *pipB2-*2HAchr	this study
AAG025scs	12023	*sifA* ^−^, *sopD2* ^−^, *pipB2-*2HAchr	this study

**Table 3:** Plasmids used in this study.

Name	Characteristics	Source
pFVP25.1	Gfpmut3A	[49]
pKD3	Chloramphenicol cassette	[36]
pKD4	Kanamycin cassette	[36]
pKD46	repA101(ts), araBp-gam-bet- exo	[36]
pCP20	ts-rep, [FLP]	[36]
pDsRed_T3.S4T	DsRed Arabinose-inducible	D. Bumann
pSopD2-2HA	SopD2-2HA in *Salmonella*	This study
pPipB2-2HA	PipB2-2HA in *Salmonella*	[22]

### Bacterial mutagenesis

Mutagenesis was carried out by using the gene disruption method described by Datsenko and Wanner, except that 10 mM arabinose was used to induce expression of the red recombinase [36]. The strains expressing PipB2-2HA, SopD2-2HA or SseJ-2HA from the chromosome were obtained using the method described by Uzzau and colleagues [37]. The oligonucleotide primers used to amplify pKD4 kanamycin or pKD3 chloramphenicol resistance genes are listed in Table 4. All mutagenesis was performed in the 12023 wild-type strain. Strains carrying two or three mutations were created by transduction using the phage P22 HT105 int. Phage-free transductants were selected for analysis. Gene deletions and transductions were checked by PCR.

**Table 4:** Oligonucleotides used in this study.

Oligonucleotides	Sequence (5′ to 3′)
SopD2 chromosomal 2-HA tag O-417	TTTATTTATAAGTGAAAAGTCGAGTTGTCGCAATATGCTTATATATCCGTACGATGTACCTGACTATGCATATCCGTACGATGTTCCAGACTATGCTTGAGTGTAGGCTGGAGCTGCTTC
SopD2 chr 2-HA tag O-114	AAAAGCGTACAAAAAAGGCTCCATATCAGTGGGGCCCATATGAATATCCTCCTTAG
PipB2 chr 2-HA tag O-418	AAGCACACAAACACTCTTTAACGAATTTTATAGTGAAAATATTTATCCGTACGATGTACCTGACTATGCATATCCGTACGATGTTCCAGACTATGCTTGAGTGTAGGCTGGAGCTGCTTC
PipB2 chr 2-HA tag O-118	ATTGCTTTTATTTCAGATTTACGTCAAAAAGGGCCATATGAATATCCTCCTTAG
SopD2 PCR check mutagenesis, O-126	GCGCCTGGCGTCGCATTC
SopD2 PCR check mutagenesis, O-127	CTCGTACCTTCACGCAGACC
PipB2 PCR check mutagenesis, O-143	CGTAAAGGGGGTATTCACCTTATCTC
PipB2 PCR check mutagenesis, O-144	GGCACTTATACATCCAGGCATAGCG

### Eukaryotic cells and culture conditions

RAW264.7 and HeLa cell lines were grown in DMEM (GibcoBRL) supplemented with 10% foetal calf serum (FCS; GibcoBRL), 2 mM nonessential amino acids, and glutamine (GibcoBRL) at 37°C in 5% CO_2_. Bone marrow cells were isolated from femurs of 6- to 10-week-old C57BL6 female mice and differentiated into macrophages as previously described [38].

### Bacterial infection and replication assays

HeLa cells were seeded in 6-well plates with or without 12-mm diameter glass coverslips at a surface ratio of 1/10, 24 h before infection. Bacteria were incubated overnight at 37°C with shaking, diluted 1∶33 in fresh LB broth, and incubated in the same conditions for 3.5 h. The cultures were diluted in Earle's buffered salt solution (pH 7.4) and added to the cells at a multiplicity of infection of 100∶1. The infection was allowed to proceed for 10 min at 37°C in 5% CO_2_. Macrophages were seeded at a density of 10^6^ cells per well in 6-well tissue culture plates 24 h before use. Bacteria were cultured overnight at 37°C with shaking and were opsonised in DMEM containing FCS and 10% normal mouse serum for 30 min on ice. Bacteria were added to the cells at a multiplicity of infection of 100∶1. Plates were centrifuged at 500 g for 5 min at 4°C and incubated for 30 min at 37°C in 5% CO_2_. Cells were washed three times with growth medium containing 100 µg/ml gentamicin and then incubated in this medium for 1 h, after which the gentamicin concentration was decreased to 10 µg/ml for the remainder of the experiment. For enumeration of intracellular bacteria, macrophages were washed three times with PBS and lysed with 0.1% Triton X-100 for 10 min, and a dilution series was plated onto LB agar plates. Plates were incubated overnight at 37°C, and colonies were counted. Each time point was performed in triplicate, and each experiment was performed three times or more.

### RNA interference

10–20% confluent HeLa cells were transfected with RNA duplexes (Quiagen) previously described [13] using siPORT Amine transfection agent (Applied Biosystems Ambion) according to the manufacturer's instructions. Cells were further incubated for 72 hours.

### Drugs and reagents

Brefeldin A (BFA, Sigma), nocodazole (Sigma), cytochalasin D (Biomol) stock solutions were prepared in dimethyl sulphoxide (DMSO) and kept at −20°C. Working concentrations were 5 µg/ml for BFA and 2µg/ml for both nocodazole and cytochalasin D. Drugs were added on the cells either at 3 h30 or 14 h p.i. for a further incubation of 4 h or 2 h respectively. Stock solution of arabinose (Sigma) was prepared (20%) in water and sterile filtered. Bacterial DsRed expression was induced by addition of arabinose to the cells (0.4%) at 12 h or 14 h post infection and cells incubated of further 4 h. Filipin (Sigma) stock solution was prepared in DMSO (25 mg/ml) and stored at −20°C. To label cellular cholesterol, fixed cells were stained for 2 h with 50 µg/ml filipin in PBS containing 10% normal foetal bovine serum (FBS, GibcoBRL).

### Immunofluorescence

Cells grown on coverslips were fixed with 3% paraformaldehyde (pH 7.4) in PBS at room temperature for 10 min. For immunostaining of tubulin, cell were further fixed in 80% methanol/1% paraformaldehyde for 5 min at −20°C. Fixed cells were washed three times in PBS and permeabilized with 0.1% saponin in PBS. Primary and secondary antibodies were diluted in PBS containing 0.1% saponin and 5% horse serum. Coverslips were incubated with primary antibodies for 60 min at room temperature, washed once in PBS containing 0.1% saponin and then incubated with appropriate secondary antibodies. Coverslips were mounted onto glass slides using ProLong Gold (Invitrogen). Cells were observed with an epifluorescence microscope (Leica) or a LSM510 confocal laser scanning microscope (Zeiss).

### Antibodies

The mouse monoclonal antibodies anti-LAMP1 H4A3 and anti-CD63 H5C6, developed by J. T. August and J. E. K. Hildreth, obtained from the Developmental Studies Hybridoma Bank (DSHB) under the auspices of the NICHD and maintained by the University of Iowa (Department of Biological Sciences, Iowa, IA), were used at a dilution of 1∶1000. The rabbit anti-LAMP1 was obtained from Dr. Minoru Fukuda (La Jolla Cancer Research Foundation) was used at a dilution of 1∶1000. The mouse monoclonal antibodies anti-LC3 (clone 5F10; Nanotools) and FK2 (which detects both mono- and polyubiquitinylated proteins) (Biomol) were used at a dilution of 1∶50 and 1∶10^4^, respectively. The rabbit anti-Sequestosome-1 (SQSTM1, also known as P62) (H-290; Santa Cruz Biotechnology) was used at a dilution of 1∶100. The rabbit anti-kinesin HC (KIF5B) antibody PCP42, generously provided by R. Vale (University of California, San Francisco), was absorbed on *Salmonella* acetone powder to remove contaminating anti-enterobacteria antibodies and used at a dilution of 1∶100. The mouse anti-HA (clone 16B12; Covance, Richmond, CA) and the rat anti-HA (clone 3F10; Roche) antibodies were used at 1∶1000 and 1∶200, respectively. The rabbit anti-Scamp3 antibody, generously provided by J. David Castle (University of Virginia, Charlottesville) was used at a dilution of 1∶250. The sheep anti human TGN46 antibody (Serotec) was used at a dilution of 1∶500. The mouse anti-tubulin (Sigma) was used at a dilution of 1∶2000. The mouse anti vATPase monoclonal antibody (OSW2) was obtained from Dr Satoshi B. Sato (Kyoto University) and was used at 1∶1000. The rabbit anti-Rab7 [39] and the mouse anti-Rab 5 (D-11, Santa Cruz Biotechnology) were used at a dilution of 1∶200 and 1∶100, respectively. The rabbit anti-calnexin (Stressgen) and the rabbit anti-cathepsin D (Dako) were used at a dilution of 1∶500 and 1∶50, respectively. The rabbit anti-mannose 6-phosphate receptor was a kind gift by Prof. Bernard Hoflack (University of Dresden) and was used at a dilution of 1∶250. Mouse anti-LBPA, generously provided by Prof. Jean Gruenberg (University of Geneva), was used at a dilution of 1∶50. The mouse (1E6, Biodesign) and rabbit anti-*Salmonella* LPS (Difco Laboratories) antibodies were used at a concentration of 1∶1000 and 1∶2000, respectively. Mouse monoclonal antibody against human MHC class II molecules (clone L243) was used at 1/10. Secondary antibodies (donkey anti-rabbit, anti-rat, anti-mouse or anti-sheep IgG conjugated to FITC, Texas red, or cyanine 5) were purchased from Jackson ImmunoResearch and used at a dilution of 1∶100. The goat anti-rabbit or anti-mouse IgG conjugated to Alexa Fluor 350 (Molecular Probes) were used at a dilution of 1∶1000. Polymerized actin was stained with Alexa 568-conjugated phalloidin (Invitrogen).

### Scoring of phenotypes by epifluorescence microscopy

SCVs were labelled by using antibodies against the lysosomal glycoprotein LAMP1. Infected cells were observed by epifluorescence, and the percentage of GFP-expressing bacteria present in a vacuole was determined by counting the total number of bacteria and the number of bacteria encircled by the LAMP1 marker. The percentage of SCVs positive for LC3, P62, FK2 or kinesin-1 was determined by visualizing GFP-expressing bacteria in the green channel, LAMP1 in the UV channel (using Alexa Fluor 350 secondary antibodies), and LC3, P62, FK2 or kinesin HC in the red channel. The percentage of bacteria expressing DsRed upon induction with arabinose was enumerated by visualizing DsRed-expression in the red channel, *Salmonella* LPS in the UV channel, and LC3, P62 or FK2 in the green channel. The percentage of infected cells containing LAMP1-tubules, PipB2-2HA- or SseJ-2HA-positive tubules was determined by counting the total number of infected cells and the number of infected cells containing the respective tubule stained. The scorings for each experiment were at least performed three times. All statistical analyses were performed by using InStat (GraphPad). The two-tailed P value was calculated.

### Quantitative analysis of confocal images

Detector settings were optimized to match PMT gain and offset to the most and the less fluorescent area of coverslips, respectively. For each experiment these settings were not changed. Two colour images of GFP-*Salmonella* (green)-infected HeLa cells immunolabeled for kinesin heavy chain (red) were recorded. Individual *Salmonella*-containing vacuoles (SCVs) were analyzed using Zeiss LSM510 software as follows. The kinesin-1 enrichment was defined as the mean fluorescence of the SCV minus the mean fluorescence of an identical adjacent cellular area. The relative amount of kinesin heavy chain enrichment of each SCV was plotted for the *sifA^−^* and *sifA^−^sopD2^−^* SCVs.

### Processing for conventional electron microscopy and scoring

Cells were fixed for 1 h at room temperature with 2.5% glutaraldehyde (Sigma) in 0.1 M cacodylate buffer, pH 7.2, containing 0.1 M sucrose, 5 mM CaCl_2_ and 5 mM MgCl_2_. Cells were then washed twice with the same buffer and postfixed for 1 h at room temperature with 1% osmium tetroxide (Electron Microscopy Sciences) in the same buffer devoid of sucrose. Cells were then scraped off the dishes and concentrated in 2% agar in the same buffer and treated for 1 h at room temperature with 1% uranyl acetate in Veronal buffer. Samples were dehydrated in a graded series of acetone and embedded in Epon resin. Thin sections were stained with 2% uranyl acetate in distilled water and then with lead citrate. 50 to 100 different vacuoles per sample, on two different EM grids, were scored as being within normal SCVs, phagolysosomes, autophagic vacuoles or within the cytosol. Care was taken to avoid serial sections.

### Mouse mixed infections

Six- to eight-week-old C57BL/6 mice were inoculated intraperitoneally with equal amounts of two bacterial strains for a total of 10^5^ bacteria per mouse. The spleens were harvested 48 h after inoculation and homogenized. Bacteria were recovered and enumerated after plating a dilution series onto LB agar and LB agar with the appropriate antibiotics. CIs were determined for each mouse [29] and a minimum of three mice were infected. The CI is defined as the ratio between the mutant and wild type strains within the output (bacteria recovered from the mouse after infection) divided by their ratios within the input (initial inoculum). Unpaired t-test analysis was performed to compare two CIs, and a one-sample t-test comparing the log of the CI to 0 was used to determine whether the CI was significantly different from 1. All statistical analyses were performed using Prism (GraphPad).
